# Impact of advanced radiotherapy techniques and dose intensification on toxicity of salvage radiotherapy after radical prostatectomy

**DOI:** 10.1038/s41598-019-57056-9

**Published:** 2020-01-10

**Authors:** Natsuo Tomita, Kaoru Uchiyama, Tomoki Mizuno, Mikiko Imai, Chikao Sugie, Shiho Ayakawa, Masanari Niwa, Tooru Matsui, Shinya Otsuka, Yoshihiko Manabe, Kento Nomura, Takuhito Kondo, Katsura Kosaki, Akifumi Miyakawa, Akihiko Miyamoto, Shinya Takemoto, Yuto Kitagawa, Takahiro Yasui, Yuta Shibamoto

**Affiliations:** 1 Department of Radiology, Nagoya City University Graduate School of Medical Sciences, 1 Kawasumi, Mizuho-cho, Mizuho-ku, Nagoya, Aichi 467-8601 Japan; 2 Department of Radiology, Kariya Toyota General Hospital, 5-15 Sumiyoshi-cho, Kariya, Aichi 448-8505 Japan; 3 Department of Radiology, Nagoya Daini Red Cross Hospital, 2-9 Myoken-cho, Showa-ku, Nagoya, Aichi 466-8650 Japan; 4 Department of Radiation Oncology, JCHO Chukyo Hospital, 1-1-10 Sanjo, Minami-ku, Nagoya, Aichi 457-8510 Japan; 5 Department of Radiation Oncology, Suzuka General Hospital, 1275-53 Yamanoue, Yasuzuka-cho, Suzuka, Mie 513-0818 Japan; 6 Department of Radiology, Konan Kosei Hospital, 137 Oomatsubara, Takaya-cho, Konan, Aichi 483-8704 Japan; 7 Department of Radiology, Okazaki City Hospital, 3-1 Goshoai, Koryuji-cho, Okazaki, Aichi 444-8553 Japan; 8 Department of Radiation Oncology, Nanbu Tokushukai Hospital, 171-1 Hokama, Yaese-cho, Shimajiri, Okinawa 901-0493 Japan; 9 Department of Radiotherapy, Nagoya City West Medical Center, 1-1-1 Hirate-cho, Kita-ku, Nagoya, Aichi 462-8508 Japan; 10 Department of Radiology, Narita Memorial Hospital, 134 Haneihonmachi, Toyohashi, Aichi 441-8029 Japan; 11 Department of Radiation Oncology, Kasugai Municipal Hospital, 1-1-1 Takaki-cho, Kasugai, Aichi 486-8510 Japan; 12 Department of Radiation Oncology, National Hospital Organization Nagoya Medical Center, 4-1-1, Sannomaru, Naka-ku, Nagoya, Aichi 460-0001 Japan; 13 Department of Radiation Oncology, Hokuto Hospital, 7-5 Kisen, Inada-cho, Obihiro, Hokkaido, 080-0833 Japan; 14 Department of Radiology, Fujieda Heisei Memorial Hospital, 123-1 Mizukami-cho, Fujieda, Shizuoka 426-8662 Japan; 15 Department of Nephro-urology, Nagoya City University Graduate School of Medical Sciences, 1 Kawasumi, Mizuho-cho, Mizuho-ku, Nagoya, Aichi 467-8601 Japan

**Keywords:** Radiotherapy, Prostate cancer

## Abstract

The safety and efficacy of dose-escalated radiotherapy with intensity-modulated radiotherapy (IMRT) and image-guided radiotherapy (IGRT) remain unclear in salvage radiotherapy (SRT) after radical prostatectomy. We examined the impact of these advanced radiotherapy techniques and dose intensification on the toxicity of SRT. This multi-institutional retrospective study included 421 patients who underwent SRT at the median dose of 66 Gy in 2-Gy fractions. IMRT and IGRT were used for 225 (53%) and 321 (76%) patients, respectively. At the median follow-up of 50 months, the cumulative incidence of late grade 2 or higher gastrointestinal (GI) and genitourinary (GU) toxicities was 4.8% and 24%, respectively. Multivariate analysis revealed that the non-use of either IMRT or IGRT, or both (hazard ratio [HR] 3.1, 95% confidence interval [CI] 1.8–5.4, *p* < 0.001) and use of whole-pelvic radiotherapy (HR 7.6, CI 1.0–56, *p* = 0.048) were associated with late GI toxicity, whereas a higher dose ≥68 Gy was the only factor associated with GU toxicities (HR 3.1, CI 1.3–7.4, *p* = 0.012). This study suggested that the incidence of GI toxicities can be reduced by IMRT and IGRT in SRT, whereas dose intensification may increase GU toxicity even with these advanced techniques.

## Introduction

Prostate cancer patients with biochemical recurrence (BCR) after radical prostatectomy (RP) have a risk of metastasis and cancer death^1^. Approximately 30% of patients with an adverse feature, such as high Gleason score, develop BCR after RP^2^. Salvage radiotherapy (SRT) is the only curative option for these patients^3,4^. The target volume of SRT is the prostate bed with or without the seminal vesicle^5^, which is situated precisely between the bladder and rectum. In a dosimetric study using consensus guidelines for target volume delineations of SRT, intensity-modulated radiation therapy (IMRT) enabled dose increase within acceptable dose constraints of the organs at risk (OARs) such as the bladder and rectum^6^. Regarding definitive radiotherapy (RT) without RP, the ratio of IMRT markedly increased in the 2000s^7^ due to benefits of high-dose prescription^8^. As systematic reviews and meta-analyses revealed that dose escalation improved biochemical control even in SRT^9,10^, dose escalation with IMRT has been employed for patients with BCR after RP^11–13^. However, dose-escalated RT, even with the IMRT technique, may increase toxicity. Image-guided radiation therapy (IGRT) enables more accurate setup by computed tomography (CT) immediately before treatment. IGRT is also one of the recent advanced RT techniques, as is IMRT, and is used more commonly with IMRT. However, the safety and efficacy of dose-escalated RT with IMRT and IGRT remain unclear. In addition, whether IMRT and IGRT reduce toxicity in SRT is still unknown. Thus, we examined the impact of advanced RT techniques and dose intensification on the toxicity of SRT after RP.

## Methods

### Patient selection

We identified 421 patients who received SRT for BCR after RP between 2005 and 2017 at 15 institutions. BCR included either PSA elevation or PSA persistence after RP. PSA elevation was defined as a PSA increase ≥0.10 ng/ml within two or more measurements^4^. PSA persistence was defined as a serum concentration ≥0.10 ng/ml at one month after RP^14^. Thus, a study cohort of 421 patients had a detectable PSA and were treated as SRT rather than adjuvant RT according to the guideline^15^. All patients had histologically confirmed adenocarcinoma of the prostate. All patients had no evidence of lymph node or other metastases on diagnostic imaging. This study was performed in accordance with the ethical standards laid down in the 1964 Declaration of Helsinki and its later amendments. As this study is a retrospective observational study, informed consent was obtained in the form of opt-out on the website. This study was approved by each institutional review board of Nagoya City University Graduate School of Medical Sciences, Kariya Toyota General Hospital, Nagoya Daini Red Cross Hospital, JCHO Chukyo Hospital, Suzuka General Hospital, Konan Kosei Hospital, Okazaki City Hospital, Nanbu Tokushukai Hospital, Nagoya City West Medical Center, Narita Memorial Hospital, Kasugai Municipal Hospital, National Hospital Organization Nagoya Medical Center, Hokuto Hospital, and Fujieda Heisei Memorial Hospital.

### Radiotherapy and androgen deprivation therapy

SRT was delivered to the prostate and seminal vesicle bed at a median (range) dose of 66 Gy (54–84) in equivalent 2-Gy fractions (EQD2). All doses in this article are expressed in EQD2. All patients were treated using 6–18 MV photon beams at conventional fractionation or moderate hypofractionation (1.8–3.0 Gy/fraction). IMRT and IGRT were used for 225 (53%) and 321 (76%) patients, respectively. Of 321 patients undergoing IGRT, kilovoltage CT and megavoltage CT were used for 180 (56%) and 141 (44%) patients, respectively. Among all 421 patients, 100 (24%), 96 (23%), and 225 (53%) patients were treated by only 3-dimensional conformal radiotherapy (3DCRT), 3DCRT with IGRT (i.e., IG-3DCRT), or IMRT with IGRT (i.e., IG-IMRT), respectively. Whole-pelvic RT (WPRT) was administered to 15 patients (4%) at a median (range) dose of 42.8 (34.2–56.4) Gy at the discretion of the attending radiation oncologists.

The dose-volume data of the rectum and bladder were analyzed, focusing on 50 patients treated at Nagoya City University Hospital, since the data were not available in most of the other institutions. The whole rectum and bladder were delineated. Among these 50 patients, 28 and 22 patients were treated by 3DCRT without IGRT and IG-IMRT, respectively. WPRT was not administered to these 50 patients.

The use of androgen deprivation therapy (ADT) combined with SRT and its duration was at the discretion of each urologist. A luteinizing hormone releasing hormone (LHRH) analog and/or anti-androgen therapy (i.e. bicalutamide) was administered to 64 patients (15%) as neoadjuvant, concurrent, and/or adjuvant ADT.

### Statistical analysis

The primary endpoint was the incidence of late gastrointestinal (GI) and genitourinary (GU) toxicity, as defined by National Cancer Institute Common Terminology Criteria for Adverse Events version 4.0^16^. Toxicities were always checked at each follow-up prospectively. A follow-up interval was 3 months in principle and was extended to every 4–6 months at 3–5 years after the start date of SRT when there was no complication with the maintenance of a low PSA level. The follow-up time was calculated from the start date of SRT. RT techniques in each era were compared by the Fisher’s exact test. SRT doses and RT techniques in each era were compared by the Student’s t-test. The distributions of toxicity were calculated by the cumulative incidence method. The significance of these outcomes was examined by the Gray’s test. Fine-Gray proportional hazards models were used in the multivariate analysis. The following variables were included in the multivariate analysis as covariates: age at SRT, time between RP and SRT, SRT dose, fraction dose, use of WPRT, and use of ADT. As for advanced RT techniques, the factor of 3DCRT without IGRT vs IG-3DCRT vs IG-IMRT was used as the covariate in the multivariate analysis. All statistical analyses were carried out using EZR^17^, which is a graphical user interface for R (version 3.4.1; R Foundation for Statistical Computing, Vienna, Austria). A *p*-value of <0.05 was defined as significant.

## Results

### Patient and treatment characteristics

The median (range) age at SRT was 69 (49–80) years. The median (range) time between RP and SRT was 17 (0–163) months. Regarding the year of SRT, 80 (19%), 184 (44%), and 157 (37%) patients underwent SRT in 2005–2009, 2010–2014, and 2015–2017, respectively. The patient and treatment characteristics are presented in Table 1. Mean doses of SRT in 2005–2009, 2010–2014, and 2015–2017 were 61.7, 65.4, and 67.4 Gy, respectively (*p* < 0.001). The proportions of IMRT in 2005–2009, 2010–2014, and 2015–2017 were 14%, 46%, and 82%, respectively (*p* < 0.001), and those of IGRT were 43%, 71%, and 100%, respectively (*p* < 0.001). The mean (range) prescribed dose in patients treated using 3DCRT vs IMRT was 62.8 (54.0–75.0) vs 67.8 (61.5–84.0) Gy, respectively (*p* < 0.001). The mean (range) prescribed dose in patients treated using non-IGRT vs IGRT was 64.3 (54.0–75.0) vs 65.8 (55.0–84.0) Gy, respectively (*p* = 0.001).

**Table 1:** Patient and treatment characteristics.

Characteristic	*n* = 421
Age at SRT (years)	69 (49–80)
**Gleason score**	
≤6	46 (11%)
7	222 (55%)
8–10	152 (36%)
**Tumor stage**	
pT2	231 (55%)
pT3a	120 (28%)
pT3b	70 (17%)
Surgical margin +	232 (55%)
Pelvic node metastasis +	9 (2%)
PSA at BCR (ng/ml)	0.36 (0.10–11.36)
Time between RP and SRT (months)	17 (0–163)
Year of SRT 2005–2009	80 (19%)
2010–2014	184 (44%)
2014–2017	157 (37%)
SRT dose in EQD2 (Gy)	66.0 (54–84)
SRT fraction dose (Gy)	2.0 (1.8–3.0)
IMRT use	225 (53%)
IGRT use	321 (76%)
WPRT use	15 (4%)
ADT use	64 (15%)

Data are n (%) or median (range).

SRT salvage radiotherapy, PSA prostate-specific antigen,

BCR biochemical recurrence,RP radical prostatectomy,

EQD2 equivalent dose in 2-Gy fractions,

IMRT intensity-modulated radiation therapy,

IGRT image-guided radiation therapy,

WPRT whole-pelvic radiotherapy,

ADT androgen deprivation therapy.

### Dosimetric analysis

**Figure 1:**
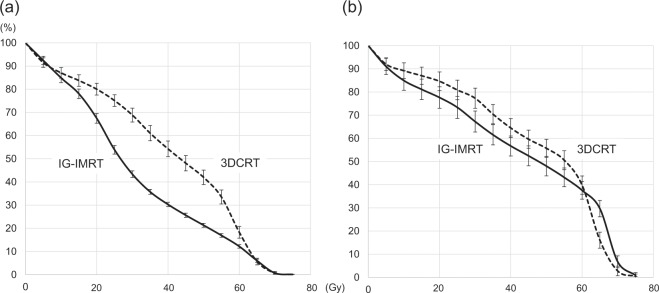
Comparison of the average dose-volume histograms (DVHs) between 3-dimensional conformal radiotherapy (3DCRT) and image-guided intensity-modulated radiation therapy (IG-IMRT) for the (**a**) rectum and (**b**) bladder. Bars represent standard errors at each dose.

Table 2 shows the dose-volume data of the rectum and bladder in 50 patients treated at Nagoya City University Hospital. Mean prescribed doses of 3DCRT and IG-IMRT were 63.6 and 68.3 Gy, respectively (*p* < 0.001). Figure 1a,b show the comparison of the average dose-volume histograms (DVHs) of the rectum and bladder between 3DCRT and IG-IMRT, respectively. IMRT reduced the rectum volume irradiated at 20–60 Gy (V20–60). However, the doses to 1% and 2% volume (D1 and D2) of the rectum were higher in the IG-IMRT group than the 3DCRT group because of the higher prescription in the IG-IMRT group. V20–60 of the bladder also tended to be lower in IG-IMRT than 3DCRT, but these differences were not significant. The high-dose areas (D1, D2, and V60) were higher in the IG-IMRT group than the 3DCRT group.

**Table 2:** Comparison of the dose-volume data with 3DCRT (n = 28) and IMRT (n = 22) among prostate cancer patients after salvage radiotherapy.

	Rectum			Bladder		
	3DCRT (%)	IMRT (%)	*p*-value	3DCRT (%)	IMRT (%)	*p*-value
V10	87.1 ± 12.2	84.9 ± 9.6	0.50	89.2 ± 17.9	84.9 ± 19.7	0.44
V20	80.0 ± 13.4	67.5 ± 9.8	<0.001	84.6 ± 21.2	77.6 ± 21.7	0.27
V30	68.9 ± 15.9	43.4 ± 6.7	<0.001	77.3 ± 23.1	67.2 ± 21.3	0.12
V40	54.3 ± 17.8	30.3 ± 4.5	<0.001	64.5 ± 21.5	56.7 ± 20.3	0.21
V50	42.0 ± 16.6	21.3 ± 3.9	<0.001	55.7 ± 20.5	48.0 ± 19.7	0.19
V60	18.3 ± 13.5	12.2 ± 3.4	0.030	39.8 ± 20.9	37.6 ± 18.0	0.69
V65	5.7 ± 7.6	6.0 ± 3.3	0.83	16.1 ± 18.3	29.7 ± 16.6	0.010
Dmean	40.2 ± 7.7	31.3 ± 2.2	<0.001	45.9 ± 11.9	43.2 ± 12.1	0.45
D50	42.6 ± 11.5	26.8 ± 3.1	<0.001	47.3 ± 18.2	42.2 ± 19.1	0.35
D2	63.7 ± 4.0	67.4 ± 2.4	<0.001	65.2 ± 4.0	70.7 ± 2.7	<0.001
D1	63.9 ± 4.0	68.2 ± 2.5	<0.001	65.4 ± 4.0	71.1 ± 2.7	<0.001

Data are mean ± standard deviations.

*3DCRT* 3-dimensional conformal radiotherapy, *IMRT* intensity-modulated radiation therapy, *V10*, *V20*, *V30*, *V40*, *V50*, *V60*, *V65* volume (%) of the rectum and the bladder receiving at least 10, 20, 30, 40, 50, 60, and 65 Gy, respectively, *Dmean* mean dose, *D1*, *D2*, *D50* dose to 1%, 2%, and 50% volume of the rectum and the bladder, respectively.

### GI and GU toxicity

**Figure 2:**
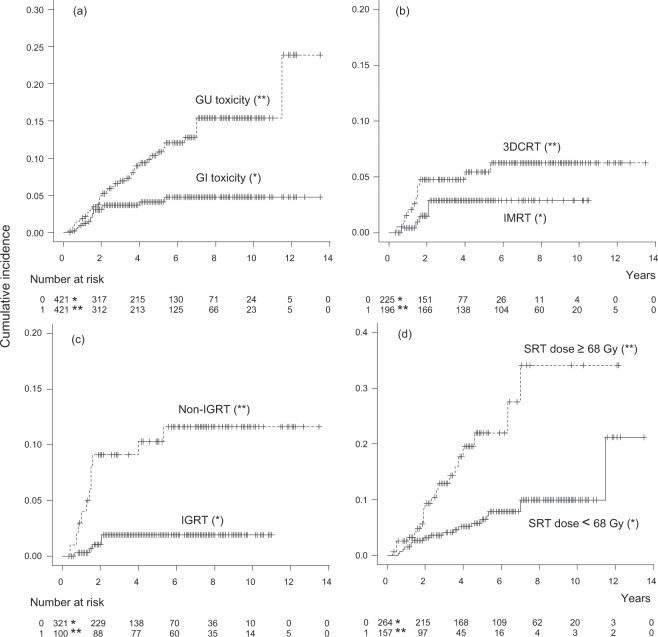
Cumulative incidence of late grade 2 or higher toxicities after salvage radiotherapy. (**a**) Late gastrointestinal (GI) and genitourinary (GU) toxicities, (**b**) comparison of GI toxicities between the 3-dimensional conformal radiotherapy (3DCRT) and intensity-modulated radiation therapy (IMRT) groups, (**c**) comparison of late GI toxicities between the image-guided radiation therapy (IGRT) and non-IGRT groups, and (**d**) comparison of late GU toxicities between the dose <68 Gy and ≥68 Gy groups.

The median (range) follow-up was 50 (4–162) months. Late grade 1, 2, and 3 GI toxicities were observed in 37 (8.8%), 6 (1.4%), and 10 patients (2.4%), respectively. Ten (2.4%) patients received argon plasma coagulation (APC) for hemorrhagic radiation proctitis. No grade 4 or higher GI toxicities were observed. The symptoms of GI toxicities are shown in Table 3. The cumulative incidence of late grade 2 or higher GI toxicities was 4.8% (95% confidence interval [CI], 2.3–7.1, Fig. 2a). Late grade 1, 2, 3, 4, and 5 GU toxicities were observed in 46 (11%), 26 (6.2%), 12 (2.9%), 1 (0.2%), and 2 (0.5%) patients, respectively. Eight patients (1.9%) received APC for hemorrhagic radiation cystis. The symptoms of GU toxicities are shown in Table 3. The cumulative incidence of late grade 2 or higher GU toxicities was 24% (95% CI, 5.6–39, Fig. 2a).

**Table 3:** Incidence of late gastrointestinal (GI) and genitourinary (GU) toxicities among 421 prostate cancer patients after salvage radiotherapy.

Symptom	Grade 1	Grade 2	Grade 3	Grade 4	Grade 5
**GI toxicity**					
Rectal hemorrhage	35 (8.3%)	6 (1.4%)	10 (2.4%)	0	0
Rectal pain	1 (0.2%)	0	0	0	0
Rectal stenosis	1 (0.2%)	0	0	0	0
**GU toxicity**					
Hematuria	32 (7.6%)	11 (2.6%)	8 (1.9%)	0	0
Urinary incontinence	11 (2.6%)	6 (1.4%)	2 (0.5%)	0	0
Urinary tract obstruction	0	4 (0.9%)	2 (0.5%)	0	1 (0.2%)
Non-infective cystitis	0	1 (0.2%)	0	1 (0.2%)	0
Urinary frequency	3 (0.7%)	4 (0.9%)	0	0	0
Urinary tract infection	0	0	0	0	1 (0.2%)

The results of the multivariate analysis predicting GI and GU toxicity after SRT are shown in Table 4. Non-use of advanced RT techniques (hazard ratio [HR] 3.1, 95% CI 1.8–5.4, *p* < 0.001) and use of WPRT (HR 7.6, CI 1.0–56, *p* = 0.048) were significantly associated with late grade 2 or higher GI toxicities. Adjusted HRs of non-use of IMRT and non-use of IGRT for GI toxicity were 4.5 (CI 1.7–12, *p* = 0.003) and 9.3 (CI 3.9–22, *p* < 0.001), respectively. The comparison of the cumulative incidence of late grade 2 or higher GI toxicities between the 3DCRT and IMRT groups (*p* = 0.16) and between the non-IGRT and IGRT groups (*p* < 0.001) is shown in Fig. 2b,c, respectively. Based on the multivariate analysis predicting GU toxicity, a higher SRT dose ≥68 Gy was the only factor associated with GU toxicities (HR 3.1, CI 1.3–7.4, *p* = 0.012). The comparison of the cumulative incidence of late grade 2 or higher GU toxicities between the dose < 68 Gy and the dose ≥68 Gy groups (*p* < 0.001) is shown in Fig. 2d.

**Table 4:** Multivariate analyses evaluating late grade 2 or higher toxicities in 421 patients after salvage radiotherapy.

	Gastrointestinal toxicity		Genitourinary toxicity	
	HR (95% CI)	*p*-value	HR (95% CI)	*p*-value
Age > 70	2.3 (0.68–8.0)	0.18	0.89 (0.44–1.8)	0.75
Time between RP and SRT ≤12 M	1.8 (0.58–5.3)	0.32	1.4 (0.68–2.7)	0.38
SRT dose (EQD2) ≥68 Gy	2.8 (0.90–8.6)	0.075	3.1 (1.3–7.4)	0.012
SRT fraction dose >2 Gy	1.1 (0.26–4.6)	0.91	1.1 (0.49–2.4)	0.85
Non-use of advanced RT techniques*	3.1 (1.8–5.4)	<0.001	0.89 (0.61–1.3)	0.55
Use of WPRT	7.6 (1.0–56)	0.048	0.75 (0.10–6.1)	0.79
Use of ADT	0.90 (0.19–4.1)	0.89	0.89 (0.38–2.1)	0.79

*HR* hazard ratio, *95% CI* 95% confidence interval, *SRT* salvage radiotherapy,

*RP* radical prostatectomy, *EQD2* equivalent dose in 2-Gy fractions,

*IMRT* intensity-modulated radiation therapy, *IGRT* image-guided radiation therapy,

*WPRT* whole-pelvic radiotherapy, *ADT* androgen deprivation therapy.

*Advanced RT techniques means IMRT or IGRT or both.

## Discussion

This large multi-institutional retrospective study showed that the prescribed dose increased significantly in SRT after RP, from 61.7 Gy in 2005–2009 to 67.4 Gy in 2015–2017. This may be partly because several reports with a high evidence level have demonstrated the improvement of biochemical control with dose escalation in SRT^9,10^. In addition, the prevalence of recent advanced RT techniques also seemed to lead to the dose escalation in SRT. Indeed, most physicians use IMRT for SRT in the United States^18^. Although dose escalation with advanced RT techniques may be the current direction of SRT^11–13^, the effects of dose escalation on toxicity remained unclear. The cumulative incidence of late ≥grade 2 GI complications was only 4.8% in our current study, whereas that after definitive RT ranged from 7% to 15%^19–21^. This may be because a lower dose of 64–70 Gy is usually used in SRT as compared with a dose of 76–80 Gy in definitive RT. Despite the low incidence of GI toxicity after SRT, additionally, IMRT could decrease GI toxicity compared with 3DCRT, as shown in Fig. 2b. This finding was in line with the results of a retrospective study in which the 5-year rate of ≥grade 2 GI toxicities was 1.9% in IMRT vs 10.2% in 3DCRT in SRT^22^. Reduction of GI complications with IMRT is expected because dosimetric studies showed that IMRT could improve the DVHs of the rectum even in SRT^6,23^.

Our current study showed that IGRT also contributed to the reduction of GI toxicity in SRT. Considering the results shown in Fig. 2b,c, IGRT may have a higher impact on the reduction of GI toxicity than IMRT. This may be partly because physicians might reduce the margin of the target volume when IGRT was used. In definitive RT, the combined use of IGRT may affect the DVHs difference as compared with the non-IGRT treatment^24^. The most significant reason may be simply the correction of the rectum position against intrafractional changes in rectal and bladder filling and digestion gas. The prostate bed is situated precisely between the bladder and rectum. In the case of SRT, the bladder and rectum lie next to each other because of the absence of the prostate. In SRT, intrafraction variations of the rectum position can affect the irradiated volume of the rectum directly more so than in definitive RT. IGRT and IMRT are often used together and have been reported to prevent GI and GU complications in post-prostatectomy RT^25^. On the other hand, in the national population-based study of RT after RP, the use of IMRT could not improve rates of severe GI and GU toxicity^26^. The authors considered one of the reasons for this finding to be the missing of information on the use of IGRT.

The cumulative incidence of late ≥grade 2 GU toxicities was 24% and a few patients developed severe (i.e. ≥grade 3) GU toxicities. Our current study showed that a SRT dose ≥68 Gy was an only significant factor associated with GU toxicities. Thus, this large multi-institutional retrospective study suggested that dose intensification might increase GU toxicity in SRT even with the advanced RT techniques. The incidences of ≥grade 2 GU toxicities varied among the reports of post-prostatectomy RT, and were between 10% and 34%^12,25,27,28^. In definitive RT, the volume of the bladder receiving high doses was a risk factor of GU toxicity^29^. The reason for the increase of GU toxicity with dose intensification may be the same as that for definitive RT. De Meerleer *et al*.^12^ reported that when 75 Gy in 37 fractions was prescribed in IMRT, ≥grade 2 GU toxicities were observed in 34% (23/68) patients and urethral stricture was observed in 7.4% (5/68). Anastomotic stricture is a common complication after RP, with a reported incidence of approximately 5–10%^30^. A higher SRT dose may affect the development of anastomotic stricture. The target volume of SRT necessarily encompasses a certain range of the bladder and an anastomosis site, which is a favored site of recurrence^5^. Therefore, the irradiated volume of an anastomosis site cannot be reduced by IMRT or IGRT. Although the GU toxicity of urinary tract obstruction was observed in only 7 patients (1.7%) in our current study, mild toxicities, such as grade 1 toxicities, were unable to be recorded. Additionally, the GU toxicities, such as urinary frequency and urinary tract infection, may have been due to anastomotic stricture. In fact, Table 2 and Fig. 1b suggested that even IMRT might not control the high-dose area of the bladder (i.e., doses around 65 Gy) in dose-escalated RT. Thus, dose intensification may increase GU toxicities even with these advanced techniques. The guideline of the Australian and New Zealand Radiation Oncology Group recommends SRT doses of 66–70 Gy to the prostate bed^31^. We also advocate that physicians must be careful in prescribing SRT doses ≥70 Gy in SRT after RP.

The extra dose to the bladder due to an inappropriate IMRT planning is also of concern. Radiation oncologists usually pay more attention to the DVH of the rectum rather than of the bladder because the definite dose constraint of the bladder is unclear, whereas the recommended dose constraint of the rectum has been reported^32,33^. Several studies have reported conflicting results for the significance of IMRT in managing adverse effects of SRT^23,34,35^. A population-based cohort study related to postoperative RT^34^ showed that the IMRT utilization increased from 33% in 2000–2004 to 78% in 2005–2007, and suggested that IMRT is associated with a lower rate of GI toxicities and a higher rate of GU complications than 3DCRT in RT after RP^34^. The authors considered one of the reasons for this finding to be that the increased use of IMRT facilitated the high-dose prescription during the study period. Thus, our finding was in line with the results of this population-based study in which higher doses were associated with GU toxicity. The long-term efficacy and toxicity of dose-escalated SRT should be evaluated in a randomized control trial (RCT)^36^.

Our study presented some relevant issues about SRT after RP. Several retrospective studies reported that elective pelvic lymph node irradiation (i.e. WPRT) improved biochemical control in selected patients^37,38^. With limited available evidence, WPRT is currently an option for patients with adverse features such as pathological nodal metastasis and Gleason score ≥8^30^. Our study suggested that WPRT increases the incidence of GI toxicity (HR 7.6, CI 1.0–56, *p* = 0.048). This was because WPRT encompasses a larger irradiated volume of the rectum than SRT to the prostate bed alone. Therefore, even when WPRT is performed in SRT, the advanced RT techniques with IGRT and IMRT may enable to escalate dose while constraining GI toxicity. The results of the RTOG 0534 phase 3 trial^39^, the first RCT investigating the efficacy of WPRT and concurrent ADT, are awaited.

No randomized data has shown that the combination of ADT potentiates the adverse effects in SRT^40^, and the same is true in our current study. Although we recognize the efficacy of adding ADT to SRT in consideration of the promising results of the recently published RCT^40^, only 15% patients received ADT combined with SRT in our current study. This was partly because of the lack of evidence for the combination of ADT in the era of our current study. Moreover, the low rate of the ADT combination may be partly due to race differences^41^ because most Asian patients with prostate cancer have a better prognosis than whites^42^. Japanese physicians might think that the combination of ADT is not necessarily needed in the setting of SRT.

Of patients who experienced grade 3 or higher GI or GU toxicities, 10 (2.4%) and 8 (1.9%) patients received the endoscopic mucosal cauterization for hemorrhagic radiation proctitis and cystis, respectively. APC can reduce bleeding and blood infusion by cauterizing mucosal telangiectasias^43^, and remedied bleeding due to hemorrhagic RT proctitis in most cases^44,45^. Therefore, this effective treatment is recommended for moderate and severe RT proctitis.

This large multi-institutional retrospective study had some limitations. First, the follow-up time was relatively limited. On the other hand, late GI and GU toxicities occasionally occurred after a median follow-up of 50 months, as shown in Fig. 2a. Additionally, the follow-up time was different between the advanced RT and conventional RT groups, and the cumulative incidence of toxicity may have become higher because of the longer follow-up time in the conventional RT group. Second, the lack of absolute information on the DVHs was previously mentioned. Despite these limitations, the findings of our study suggested the impact of advanced RT techniques and dose intensification on the toxicity of SRT after RP.

In conclusion, this study demonstrated that the SRT dose increased significantly with increasing use of IMRT and IGRT from 2004 to 2017. Our results suggested that the incidence of GI toxicity can be reduced by IMRT and IGRT for SRT after RP, whereas high-dose SRT may increase the incidence of GU toxicity even with these advanced techniques. These findings will facilitate the design of a clinical trial for SRT after RP.

## Data Availability

Data sharing is not applicable to this article due to ethical reasons.

## References

[1] Freedland SJ (2005). Risk of prostate cancer-specific mortality following biochemical recurrence after radical prostatectomy. JAMA.

[2] Stephenson AJ (2006). Preoperative nomogram predicting the 10-year probability of prostate cancer recurrence after radical prostatectomy. J. Natl Cancer Inst..

[3] Cornford P (2017). EAU-ESTRO-SIOG Guidelines on Prostate Cancer. Part II: Treatment of Relapsing, Metastatic, and Castration-Resistant Prostate Cancer. Eur. Urol..

[4] Mohler JL (2016). Prostate Cancer, Version 1.2016. J. Natl Compr. Canc Netw..

[5] Sidhom MA (2008). Post-prostatectomy radiation therapy: consensus guidelines of the Australian and New Zealand Radiation Oncology Genito-Urinary Group. Radiother. Oncol..

[6] Harrison A (2011). Potential for dose escalation in the postprostatectomy setting with intensity-modulated radiation therapy: a dosimetric study using EORTC consensus guidelines for target volume contours. Pract. Radiat. Oncol..

[7] Sheets NC (2012). Intensity-modulated radiation therapy, proton therapy, or conformal radiation therapy and morbidity and disease control in localized prostate cancer. JAMA.

[8] Viani GA, Stefano EJ, Afonso SL (2009). Higher-than-conventional radiation doses in localized prostate cancer treatment: A meta-analysis of randomized, controlled trials. Int. J. Radiat. Oncol. Biol. Phys..

[9] King CR (2016). The dose-response of salvage radiotherapy following radical prostatectomy: A systematic review and meta-analysis. Radiother. Oncol..

[10] Ohri N, Dicker AP, Trabulsi EJ, Showalter TN (2012). Can early implementation of salvage radiotherapy for prostate cancer improve the therapeutic ratio? A systematic review and regression meta-analysis with radiobiological modelling. Eur. J. Cancer.

[11] Cheng JC, Schultheiss TE, Nguyen KH, Wong JY (2008). Acute toxicity in definitive versus postprostatectomy image-guided radiotherapy for prostate cancer. Int. J. Radiat. Oncol. Biol. Phys..

[12] De Meerleer G (2008). Salvage intensity-modulated radiotherapy for rising PSA after radical prostatectomy. Radiother. Oncol..

[13] Ost P (2011). High-dose salvage intensity-modulated radiotherapy with or without androgen deprivation after radical prostatectomy for rising or persisting prostate-specific antigen: 5-year results. Eur. Urol..

[14] Mottet N (2017). EAU-ESTRO-SIOG Guidelines on Prostate Cancer. Part 1: Screening, Diagnosis, and Local Treatment with Curative Intent. Eur. Urol..

[15] Thompson IM (2013). Adjuvant and salvage radiotherapy after prostatectomy: AUA/ASTRO Guideline. J. Urol..

[16] Common Terminology Criteria for Adverse Events (CTCAE) Version 4.0 Available from, http://www.jcog.jp/doctor/tool/CTCAEv4J_20170912_v20_1.pdf (accessed September 11 2018).

[17] Kanda Y (2013). Investigation of the freely available easy-to-use software ‘EZR’ for medical statistics. Bone Marrow Transpl..

[18] Showalter TN (2011). Physician beliefs and practices for adjuvant and salvage radiation therapy after prostatectomy. Int. J. Radiat. Oncol. Biol. Phys..

[19] Sharma NK (2011). Intensity-modulated radiotherapy reduces gastrointestinal toxicity in patients treated with androgen deprivation therapy for prostate cancer. Int. J. Radiat. Oncol. Biol. Phys..

[20] Takemoto S (2019). Long-term results of intensity-modulated radiotherapy with three dose-fractionation regimens for localized prostate cancer. J. Radiat. Res..

[21] Tomita N (2016). High-dose radiotherapy with helical tomotherapy and long-term androgen deprivation therapy for prostate cancer: 5-year outcomes. J. Cancer Res. Clin. Oncol..

[22] Goenka A (2011). Improved toxicity profile following high-dose postprostatectomy salvage radiation therapy with intensity-modulated radiation therapy. Eur. Urol..

[23] Koontz BF (2009). Dosimetric and radiobiologic comparison of 3D conformal versus intensity modulated planning techniques for prostate bed radiotherapy. Med. Dosim..

[24] Crehange G (2012). Clinical impact of margin reduction on late toxicity and short-term biochemical control for patients treated with daily on-line image guided IMRT for prostate cancer. Radiother. Oncol..

[25] Nath SK (2010). Toxicity analysis of postoperative image-guided intensity-modulated radiotherapy for prostate cancer. Int. J. Radiat. Oncol. Biol. Phys..

[26] Sujenthiran A (2018). Treatment-related toxicity in men who received Intensity-modulated versus 3D-conformal radiotherapy after radical prostatectomy: A national population-based study. Radiother. Oncol..

[27] Tomita N (2009). Early salvage radiotherapy for patients with PSA relapse after radical prostatectomy. J. Cancer Res. Clin. Oncol..

[28] Feng M (2007). Predictive factors for late genitourinary and gastrointestinal toxicity in patients with prostate cancer treated with adjuvant or salvage radiotherapy. Int. J. Radiat. Oncol. Biol. Phys..

[29] Viswanathan AN, Yorke ED, Marks LB, Eifel PJ, Shipley WU (2010). Radiation dose-volume effects of the urinary bladder. Int. J. Radiat. Oncol. Biol. Phys..

[30] Herschorn S, Elliott S, Coburn M, Wessells H, Zinman L (2014). SIU/ICUD Consultation on Urethral Strictures: Posterior Urethral Stenosis After Treatment of Prostate Cancer. Urology.

[31] Lieng H (2018). Radiotherapy for recurrent prostate cancer: 2018 Recommendations of the Australian and New Zealand Radiation Oncology Genito-Urinary group. Radiother. Oncol..

[32] Michalski JM, Gay H, Jackson A, Tucker SL, Deasy JO (2010). Radiation dose-volume effects in radiation-induced rectal injury. Int. J. Radiat. Oncol. Biol. Phys..

[33] Tomita N (2013). Preliminary analysis of risk factors for late rectal toxicity after helical tomotherapy for prostate cancer. J. Radiat. Res..

[34] Crandley EF (2014). Treatment-related complications of radiation therapy after radical prostatectomy: comparative effectiveness of intensity-modulated versus conformal radiation therapy. Cancer Med..

[35] Goldin GH (2013). Comparative effectiveness of intensity-modulated radiotherapy and conventional conformal radiotherapy in the treatment of prostate cancer after radical prostatectomy. JAMA Intern. Med..

[36] Ghadjar P (2015). Acute Toxicity and Quality of Life After Dose-Intensified Salvage Radiation Therapy for Biochemically Recurrent Prostate Cancer After Prostatectomy: First Results of the Randomized Trial SAKK 09/10. J. Clin. Oncol..

[37] Moghanaki D (2013). Elective irradiation of pelvic lymph nodes during postprostatectomy salvage radiotherapy. Cancer.

[38] Ramey SJ (2018). Multi-institutional Evaluation of Elective Nodal Irradiation and/or Androgen Deprivation Therapy with Postprostatectomy Salvage Radiotherapy for Prostate Cancer. Eur. Urol..

[39] RTOG 0534; A phase III trial of short term androgen deprivation with pelvic lymph node or prostate bed only radiotherapy in prostate cancer patients with a rising PSA after radical prostatectomy. Available from, http://rpc.mdanderson.org/rpc/credentialing/files/0534.pdf (accessed March 21, 2019)

[40] Shipley WU (2017). Radiation with or without Antiandrogen Therapy in Recurrent Prostate Cancer. N. Engl. J. Med..

[41] Kimura T (2012). East meets West: ethnic differences in prostate cancer epidemiology between East Asians and Caucasians. Chin. J. Cancer.

[42] Robbins AS (2007). Differences in prognostic factors and survival among white and Asian men with prostate cancer, California, 1995–2004. Cancer.

[43] Takemoto S (2012). Treatment and prognosis of patients with late rectal bleeding after intensity-modulated radiation therapy for prostate cancer. Radiat. Oncol..

[44] Tucker SL (2012). Do intermediate radiation doses contribute to late rectal toxicity? An analysis of data from radiation therapy oncology group protocol 94–06. Int. J. Radiat. Oncol. Biol. Phys..

[45] Chennupati SK (2014). Late toxicity and quality of life after definitive treatment of prostate cancer: redefining optimal rectal sparing constraints for intensity-modulated radiation therapy. Cancer Med..

